# Designing HIV Testing Algorithms Based on 2015 WHO Guidelines Using Data from Six Sites in Sub-Saharan Africa

**DOI:** 10.1128/JCM.00962-17

**Published:** 2017-09-25

**Authors:** Cara S. Kosack, Leslie Shanks, Greet Beelaert, Tumwesigye Benson, Aboubacar Savane, Anne Ng'ang'a, André Bita, Jean-Paul B. N. Zahinda, Katrien Fransen, Anne-Laure Page

**Affiliations:** a Médecins sans Frontières, Amsterdam, Netherlands; b Institute of Tropical Medicine, Antwerp, Belgium; c Ministry of Health Uganda, Kampala, Uganda; d Laboratoire National de Reference, Conakry, Guinea; e National AIDS and Sexually Transmitted Infections Control Programme, Nairobi, Kenya; f Regional Delegation of Public Health for the Littoral Region, Yaounde, Cameroon; g Programme National de Lutte contre le Sida et les IST (PNLS), Bukavu, Democratic Republic of Congo; h Epicentre, Paris, France; Rhode Island Hospital

**Keywords:** WHO guidelines, diagnostic accuracy, diagnostic algorithms, human immunodeficiency virus, positive predictive value, rapid tests

## Abstract

Our objective was to evaluate the performance of HIV testing algorithms based on WHO recommendations, using data from specimens collected at six HIV testing and counseling sites in sub-Saharan Africa (Conakry, Guinea; Kitgum and Arua, Uganda; Homa Bay, Kenya; Douala, Cameroon; Baraka, Democratic Republic of Congo). A total of 2,780 samples, including 1,306 HIV-positive samples, were included in the analysis. HIV testing algorithms were designed using Determine as a first test. Second and third rapid diagnostic tests (RDTs) were selected based on site-specific performance, adhering where possible to the WHO-recommended minimum requirements of ≥99% sensitivity and specificity. The threshold for specificity was reduced to 98% or 96% if necessary. We also simulated algorithms consisting of one RDT followed by a simple confirmatory assay. The positive predictive values (PPV) of the simulated algorithms ranged from 75.8% to 100% using strategies recommended for high-prevalence settings, 98.7% to 100% using strategies recommended for low-prevalence settings, and 98.1% to 100% using a rapid test followed by a simple confirmatory assay. Although we were able to design algorithms that met the recommended PPV of ≥99% in five of six sites using the applicable high-prevalence strategy, options were often very limited due to suboptimal performance of individual RDTs and to shared falsely reactive results. These results underscore the impact of the sequence of HIV tests and of shared false-reactivity data on algorithm performance. Where it is not possible to identify tests that meet WHO-recommended specifications, the low-prevalence strategy may be more suitable.

## INTRODUCTION

The HIV rapid diagnostic tests (RDTs) are the main diagnostic tools for HIV screening and diagnosis in resource-constrained settings (1). Given the potential for the severe medical, psychological, and social impacts of HIV misdiagnosis and the evidence of elevated false-positive results from some settings, it is imperative that HIV diagnosis is confirmed to be both sensitive and specific (2).

**FIG 1:**
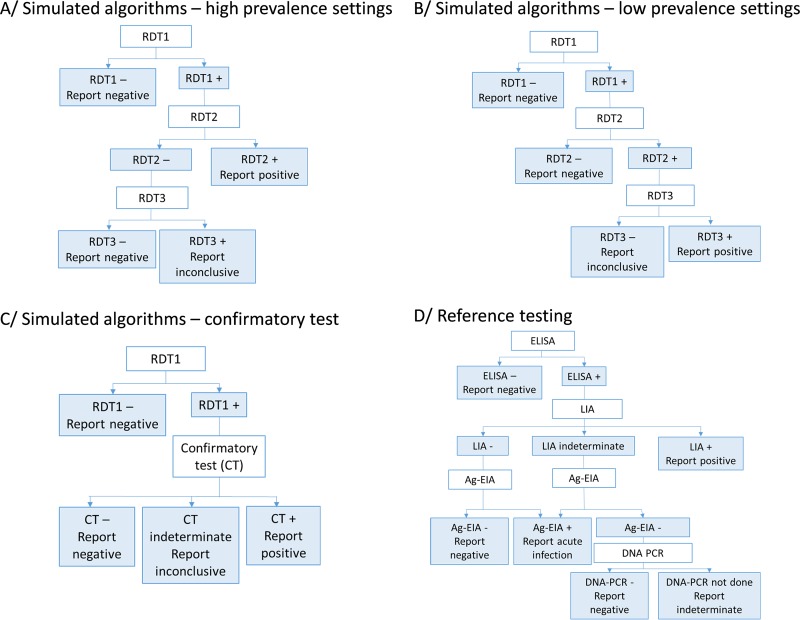
HIV testing strategies used to simulate algorithms (A to C) and reference testing algorithm used in this study (D).

In 2012 and 2015, the World Health Organization (WHO) published revisions of the HIV testing guidelines with different recommendations for low (<5%)- and high (≥5%)-HIV-prevalence settings (1, 3, 4). These recommendations call for the sequential use of up to three different serological assays, including RDTs, for final HIV diagnoses. Whereas a first nonreactive test result is sufficient to provide a final negative result in both settings, two and three reactive assays are needed to provide final HIV-positive results in high- and low-prevalence settings, respectively (Fig. 1). The guidelines stipulate that each of the three RDTs should have a sensitivity of at least 99%, while the first RDT should have at least 98% specificity and the second and third RDTs at least 99% specificity; overall, the combination should be designed to minimize the potential for shared false reactivity. Different strategies for high- and low-prevalence settings were developed based on mathematical models using three theoretical assays assumed to meet the criteria described above to achieve an overall positive predictive value (PPV) of at least 99% (1). To date, however, these recommendations and the performance of the resulting algorithms have not been validated using real data from different field contexts.

Several factors could influence the design and performance of these algorithms. Although WHO-prequalified HIV RDTs met the minimum recommended sensitivity and specificity criteria in the prequalification evaluations, several reports from different countries indicate much poorer performance in real-world settings (5–12). Moreover, little is known about shared false-reactivity results among different RDTs (13). The use of the same antigen (Ag) preparations to produce different tests, which is occurring with increasing frequency due to rebranding or relabeling arrangements among test manufacturers (1), can lead to shared cross-reactivity, though this may not be the only cause. Even low levels of shared cross-reactivity, or marginally substandard performance of one RDT, could have a meaningful impact on the performance of an algorithm.

Given concerns about false positivity raised by previous findings, over the period of 2011 to 2015 we conducted an evaluation of eight HIV RDTs and two simple confirmatory assays differentiating antibodies against several viral proteins (14). We used specimens collected at six HIV testing and counseling (HTC) centers in sub-Saharan Africa, the region most highly affected by HIV/AIDS, with approximately 70% of the total number of people living with HIV worldwide (15). Consistent with the aforementioned reports (5–12), this study revealed lower-than-expected specificity for most of the tests and important variations by specimen origin (14). Here, we have used these data to validate the performance of simulated algorithms developed according to the latest WHO recommendations. Additionally, we explored the possibility of using algorithms incorporating simple confirmatory assays that could be suitable for use in low- and middle-income countries.

## RESULTS

From August 2011 to January 2015, a total of 2,785 samples collected at the six HTC sites (comprising between 437 and 500 samples at each site) were sent to the reference laboratory. The HIV positivity rate by site ranged from 8.0% to 37.1% (Table 1). More information on the characteristics of clients included in the study are provided elsewhere (16). Using the reference algorithm, 1,306 were classified as HIV-positive clients (including 1 positive for HIV-2) and 1,474 as HIV-negative clients. Three samples with inconclusive reference results and two samples with reference results suggestive of acute infection were excluded from the analysis.

**TABLE 1:** Demographic and clinical characteristics by study site

Parameter	Value(s)						
	Guinea (Conakry)	Cameroon (Douala)	Uganda (Kitgum)	Kenya (Homa Bay)	Uganda (Arua)	DRC (Baraka)	Total
Tested at site during study period							
Total *N*	2,033	1,239	3,159	1,003	2,971	3,610	14,015
Positive on site, *n* (%)	574 (28.2)	396 (32.0)	332 (10.5)	372 (37.1)	386 (13.0)	288 (8.0)	2,348 (16.8)
Included in the study							
Total *N*	446	462	437	500	443	497	2,785
Positive, *n* (%)	222 (49.8)	214 (46.3)	213 (48.7)	224 (44.8)	212 (47.9)	221 (44.5)	1,306 (46.9)
Negative, *n* (%)	224 (50.2)	247 (53.5)	222 (50.8)	276 (55.2)	230 (51.9)	275 (55.3)	1,474 (52.9)
Acute infection, *n* (%)	0 (0)	0 (0)	2 (0.5)	0 (0)	0 (0)	0 (0)	2 (0.1)
Indeterminate, *n* (%)	0 (0)	1 (0.2)	0 (0)	0 (0)	1 (0.2)	1 (0.2)	3 (0.1)
Age and sex							
Median age, yrs (IQR)	29 (22–39)	31 (25–41)	30 (24–39)	30 (23–40)	29 (23–37)	30 (23–39)	30 (24–39)
Males, *n* (%)	132 (29.6)	163 (35.3)	176 (40.3)	201 (40.2)	213 (48.2)	177 (35.6)	1,062 (38.2)

The performance of the HIV RDTs and simple confirmatory tests assessed individually and by origin of specimens is described elsewhere (14). Of a total of 438 specimens that gave at least one false reactive result, the majority gave a falsely reactive result with only one of the eight RDTs (*n* = 295), 81 with two RDTs, 41 with three RDTs, 15 with four RDTs, 4 with five RDTs, and 2 with six RDTs. All RDTs exhibited some shared false-reactivity results with each of the seven other RDTs, with the exception of SD Bioline and Stat-Pak (Table 2).

**TABLE 2:** Number and proportion of shared falsely reactive results using RDT1 followed by RDT2

RDT1	Total no. of falsely reactive results by RDT1	No. (%) of falsely reactive results by RDT1 only^*b*^	No. (%) of falsely reactive results with RDT2^*a*^							
			Determine	Uni-Gold	Genie Fast	Vikia	Stat-Pak	Insti	SD Bioline	First Response
Determine	124	42 (33.9)		11 (8.9)	26 (21.0)	46 (37.1)	6 (4.8)	29 (23.4)	9 (7.3)	23 (18.6)
Uni-Gold	39	11 (28.2)	11 (28.2)		10 (25.6)	4 (10.3)	1 (2.6)	18 (46.2)	5 (12.8)	5 (12.8)
Genie Fast	102	46 (45.1)	26 (25.5)	10 (9.8)		17 (16.7)	6 (5.9)	25 (24.5)	8 (7.8)	19 (18.6)
Vikia	61	11 (18.0)	46 (75.4)	4 (6.5)	17 (27.9)		6 (9.8)	15 (25.6)	3 (4.9)	10 (16.4)
Stat-Pak	10	3 (30.0)	6 (60.0)	1 (10.0)	6 (60.0)	6 (60.0)		4 (40.0)	0 (0.0)	2 (20.0)
Insti	151	86 (57.0)	29 (19.2)	18 (11.9)	25 (16.6)	15 (9.9)	4 (2.7)		18 (11.9)	18 (11.9)
SD Bioline	43	9 (20.9)	9 (20.9)	5 (11.6)	8 (18.6)	3 (7.0)	0 (0.0)	18 (41.9)		20 (46.5)
First Response	142	87 (61.3)	23 (16.2)	5 (3.5)	19 (13.4)	10 (7.0)	2 (1.4)	18 (12.7)	20 (14.1)	

a The percentages in parentheses indicate the proportions of falsely reactive results by RDT2 among the samples with falsely reactive results by RDT1.

b The percentages in parentheses indicate the proportion of falsely reactive results by RDT1 that did not show any falsely reactive results with any other RDT.

For only one site, Conakry (Guinea), could we identify at least two RDTs to be used as a second or third test with sensitivity and specificity estimates of ≥99%, as recommended by WHO. Using the testing strategy for high-prevalence settings with Determine as the first test and these assays as second and third tests, the PPV of the algorithms ranged from 98.3% to 100% (Table 3). For three other sites (Douala, Cameroon; Kitgum, Uganda; Homa Bay, Kenya), only one test met the WHO criteria, necessitating the use of tests with a specificity of ≥98% as RDT2 and RDT3 and resulting in PPVs ranging from 92.7% to 100%. For the remaining two sites (Arua, Uganda; Baraka, Democratic Republic of Congo [DRC]), one test met the WHO criteria, but all others had specificities of <98%, necessitating the use of tests with specificities between 96% and 98%. The PPV of the resulting algorithms ranged from 75.8% to 99.6%. Detailed results are presented in Table 3.

**TABLE 3:**
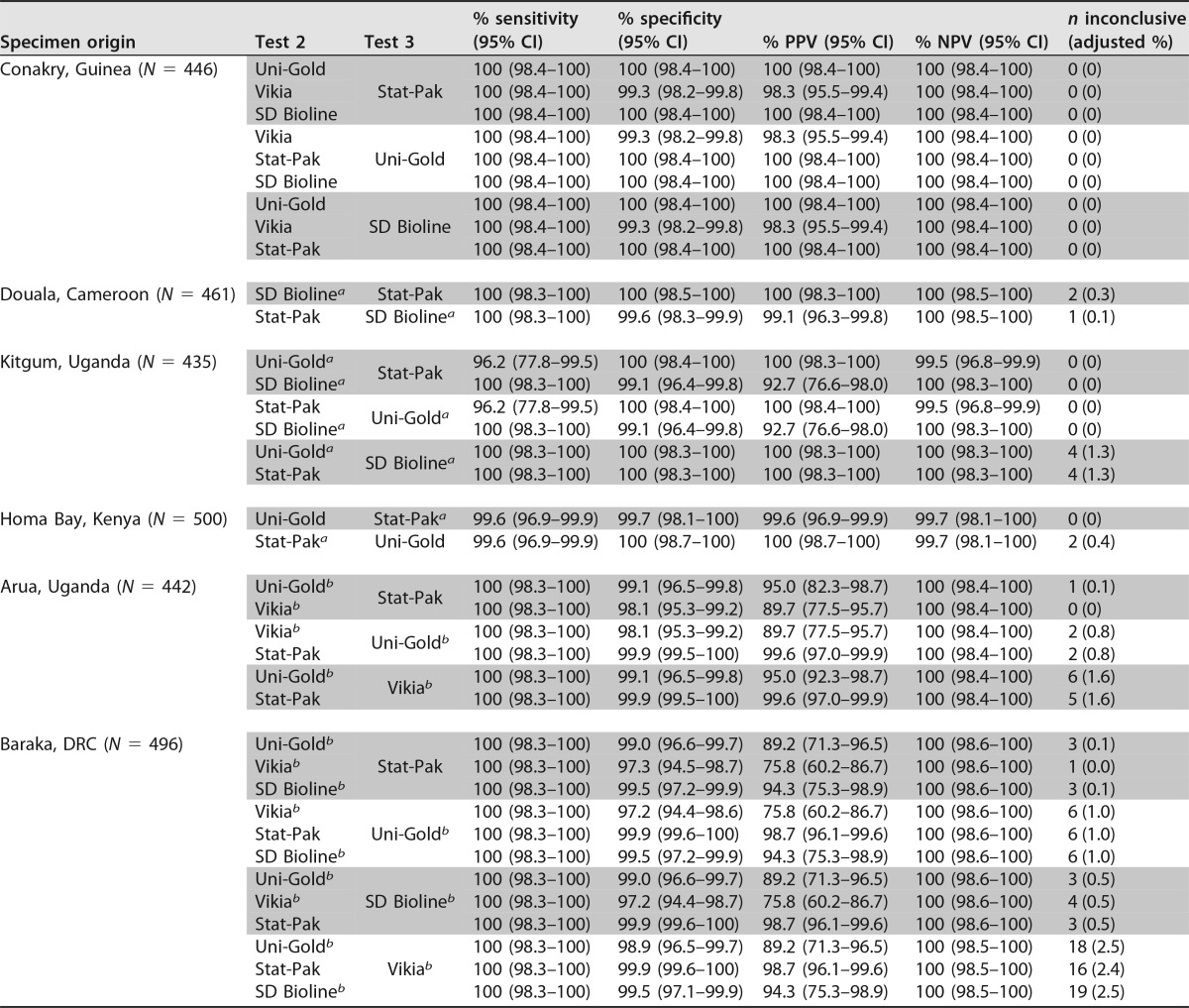
Simulated algorithms with Determine HIV-1/2 combined with other HIV RDTs in a serial 3-test algorithm for high (≥5%)-prevalence settings

^*a*^ RDT specificity was estimated to be between 98.0% and 98.9% for this site.

^*b*^ RDT specificity was estimated to be between 96.0% and 97.9% for this site.

Using the WHO strategy for low-prevalence settings, most simulated algorithms showed PPVs of ≥99%, even for the two sites (Arua, Uganda; Baraka, DRC) where tests with specificities between 96% and 98% were included in the algorithms (Table 4). The proportion of inconclusive results remained low at <1% for most algorithms but rose to 2.5% at sites where tests with specificities between 96% and 98% were included in the algorithms.

**TABLE 4:**
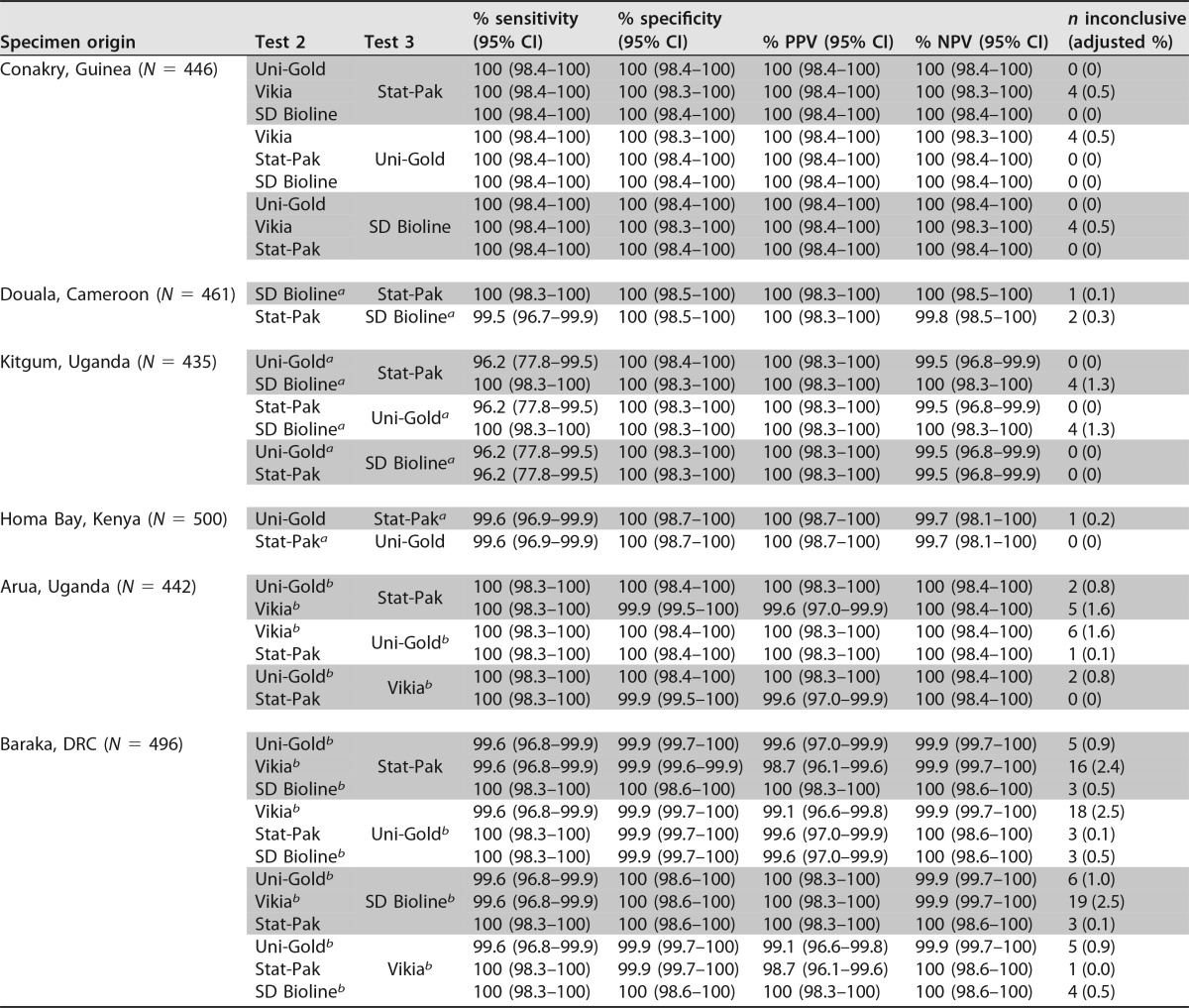
Simulated algorithms with Determine HIV-1/2 combined with other HIV RDTs in a serial 3-test algorithm for low (<5%)-prevalence settings

^*a*^ RDT specificity was estimated to be between 98.0% and 98.9% for this site.

^*b*^ RDT specificity was estimated to be between 96.0% and 97.9% for this site.

We also evaluated a simplified version of a reference algorithm, using a rapid test meeting criteria for RDT1 as a screening assay followed by a simple confirmatory assay. The PPVs of these algorithms ranged from 98.1% to 100%, with the proportions of inconclusive results ranging from 0% to 0.5% (Table 5).

**TABLE 5:**
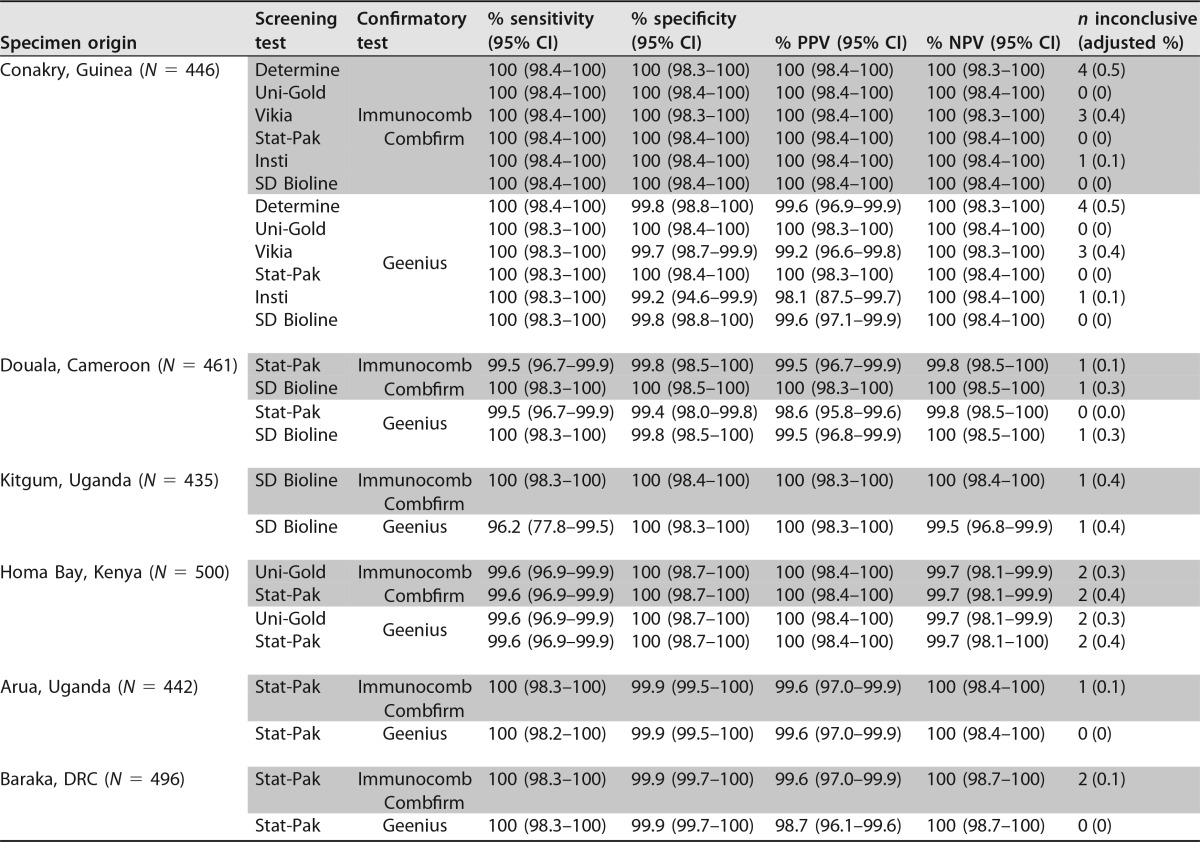
Simulated algorithms with a rapid test used as a screening test followed by a simple confirmatory test for reactive samples

## DISCUSSION

WHO-recommended HIV testing strategies were developed based on models using theoretical RDTs with high sensitivity and specificity and no shared cross-reactivity. Here, we have used the results of a large multicenter evaluation of individual RDTs to estimate the performance of HIV testing algorithms using real data from six sub-Saharan African HTC sites. To our knowledge, this was the first study that evaluated the performance of algorithms based on the new WHO recommendations; all other such studies published to date focused on strategies using either two tests or three tests, with the third test used as a tiebreaker (7, 9, 11, 17–20). Though WHO has never recommended the use of a tiebreaker due to the associated risk of generating false-positive results, this strategy is still widely used (21).

The use of several algorithms simulated here based on the strategy for high-prevalence settings resulted in a PPV of <99%, even though RDTs with high specificity were used as second and third tests, due to shared falsely reactive results among the tests used. In particular, a general trend of shared falsely reactive results between Determine and Vikia could explain the finding that combinations using these two tests with samples from Conakry resulted in a suboptimal PPV of 98.3%, despite the fact that each test used at that site had an estimated specificity of ≥99%. Although we could not identify a similar trend of shared falsely reactive results between Determine and SD Bioline, the level of shared false reactivity was high with samples from Kitgum, leading to a PPV of only 92.7% for algorithms using these tests for Kitgum despite the acceptable specificity of SD Bioline (98.6%) on specimens from this site. A larger sample size is needed to investigate whether this represents a local phenomenon or a random occurrence. In the absence of reliable knowledge on the source of antigen preparations and of a good understanding of the mechanisms underlying falsely reactive results, only raw data from RDT evaluation studies using samples from local sites can provide the necessary information to avoid shared falsely reactive results.

For sites where only one test had a specificity of >99% and tests with specificities of 96% to 98% had to be included in the algorithms, the PPV of algorithms using the strategy for high-prevalence settings varied widely depending on the order of the second and third tests. In both sites (Arua, Uganda; Baraka, DRC), only algorithms using the highly specific test Stat-Pak as the second test reached or approached the threshold, while all other combinations gave PPVs below 95%. These results underscore the importance of the order of the RDTs in the algorithm and of using the test with the highest specificity as the second (and not third) test in employing a three-test strategy in the absence of two highly specific tests.

The strategy recommended for low-prevalence settings, which requires three reactive RDTs to establish a diagnosis of HIV infection, generally resulted in algorithms with very high PPVs. For Baraka, DRC, where none of the high-prevalence algorithms achieved a PPV of ≥99%, this was the only strategy that reached the threshold. In addition, since this strategy classifies discordant results (e.g., RDT1-positive and RDT2-negative [RDT1^+^ RDT2^−^] results) as negative results, it is important to ensure that the negative predictive value (NPV), together with the PPV, is >99%, as was the case for the algorithms simulated here. This suggests that the low-prevalence-HIV testing strategy may be suitable for use not only in settings with low HIV prevalence but wherever HIV RDTs are known to have specificity issues.

We also propose a testing strategy that, similarly to a reference algorithm, relies on a sensitive screening assay followed by a simple confirmatory assay. One of these confirmatory assays, the ImmunoComb Combfirm, has shown good correlation with Western blotting in evaluations in the DRC and Ethiopia to confirm a two-RDT algorithm positive result but is no longer produced (11, 22). Another option, the Geenius assay, has generally shown performance results sufficient for recommending it as an alternative to existing confirmatory assays such as Western blotting or immunoblotting (23–29). However, here we found that the use of these confirmatory assays did not consistently ensure PPVs of ≥99% in the different combinations tested, particularly for the two sites where RDTs showed high levels of false reactivity. Given the added complexity and cost of the Geenius confirmatory assay, we conclude that it does not compare favorably with the three-RDT combination recommended by WHO for use in these settings.

One of the limitations of this study was that Determine was used as the first assay in all algorithms that we simulated. We used Determine for the same reasons for which it is currently used as the first test in most algorithms: its relative low cost and very high sensitivity. Another limitation is that our sampling strategy underrepresented clients with negative results according to the onsite algorithm, resulting in a collection of specimens that was not representative of the population screened. To account for this verification bias, we conducted a weighted analysis aimed at mitigating its effect. The inclusion of all specimens with inconclusive results from onsite testing might also explain the high proportion of falsely reactive specimens in this study compared to other evaluations, including those for WHO prequalification. We believe, however, that these data reflect the reality of HIV testing at HTC sites. Nevertheless, although centralized testing in a reference laboratory had advantages for standardization and comparison of results, it had the disadvantage of not reproducing all aspects of field conditions. In particular, we could not reproduce repeat testing for clients with inconclusive results, which might have an impact on the final performance of these algorithms. Finally, we did not illustrate the use of these algorithms in low-prevalence settings, since all specimens came from sites that would be classified as high-prevalence sites. A simple calculation using the sensitivity and specificity reported here, together with the prevalence in the setting of interest, could provide useful information on the expected PPV for such settings. In addition, since most of the low-prevalence algorithms achieved a PPV of 100%, which would not be affected by HIV prevalence, our data support the use of the recommended strategy for these settings.

This attempt to illustrate the process and results of designing an HIV testing strategy using real data offers important lessons for navigating the various obstacles in the process. First, our data underscore the impact of shared false-reactivity results on the performance of algorithms and show that this phenomenon affects most RDT combinations to different degrees. More-transparent information from test manufacturers on possible shared false reactivity due to test rebranding or common sources of antigens is needed. Moreover, shared falsely reactive results from other studies performed using a standard panel for the evaluation of different assays would provide useful complementary information. Second, our results demonstrate that data from local evaluations are important for assessing diagnostic accuracy in the specific setting, although obtaining such information is often not feasible (30). We also highlight the importance of the order of tests, particularly in using the strategy for settings of high HIV prevalence, where the test with highest specificity should be used as the second rather than the third assay. Finally, if sufficient information is available and these steps are followed, good RDT-based HIV testing algorithms can be designed, though sometimes only with the strategy recommended for low-prevalence settings.

## MATERIALS AND METHODS

### Study setting.

The samples used for this study were collected at the following voluntary or provider-initiated HTC service programs in six public health care clinics and hospitals in Sub-Saharan Africa between August 2011 and January 2015: the Centre Communautaire Matam in Conakry, Guinea; Madi Opei Clinic and Kitgum Matidi Clinic in Kitgum, Uganda; Homa Bay District Hospital in Homa Bay, Kenya; Arua District Hospital in Arua, Uganda; Nylon Hospital in Doula, Cameroon; and Baraka Hospital in Baraka, South Kivu, DRC. The details of the HIV testing algorithm used at each site are provided elsewhere (16). Minimums of 220 positive and 220 negative specimens, as classified by the algorithm used on site, were prospectively collected as described previously (16). All frozen plasma samples were then sent to the AIDS reference laboratory at the Institute for Tropical Medicine (ITM), Antwerp, Belgium, for characterization with a standard reference algorithm (Fig. 1) and for testing with eight RDTs and two simple confirmatory assays.

### Reference method for HIV diagnosis.

All plasma samples were tested at ITM using a fourth-generation enzyme-linked immunosorbent assay (ELISA) detecting both antibodies and antigens (Vironostika HIV Uni-Form II Ag/Ab; bioMérieux, France) followed by a line immunoassay (LIA) (i.e., INNO-LIA HIV I/II Score; Innogenetics NV, Ghent, Belgium) and an antigen-enzyme immunoassay (Ag-EIA) (i.e., Innotest HIV Antigen MAb; Innogenetics NV, Ghent, Belgium) and in-house DNA PCR when applicable, as described for Fig. 1.

### HIV rapid diagnostic tests.

All eight HIV RDTs (Determine HIV-1/2 [Determine; Alere, USA], Uni-Gold HIV [Uni-Gold; Trinity Biotech, Ireland], Genie Fast HIV 1/2 [Genie Fast; BioRad Laboratories, USA], Vikia HIV 1/2 [Vikia; bioMérieux, France], HIV 1/2 Stat-Pak [Stat-Pak; Chembio, USA], Insti HIV-1/HIV-2 antibody test [Insti; bioLytical, Canada], SD Bioline HIV 1/2 3.0 [SD Bioline; Standard Diagnostics, South Korea], and First Response HIV Card Test1-2.O [First Response; PMC, India]) and two simple confirmatory assays (ImmunoComb II HIV 1&2 CombFirm [ImmunoComb Combfirm; Orgenics, Alere, Israel] and Geenius HIV 1/2 confirmatory assay [Geenius; Bio-Rad, USA]) were performed at ITM on all collected plasma samples from the six study sites, as reported elsewhere (14). All tests were performed by six trained laboratory technicians. Each test was read by two technicians, each of whom was blind to the results reported by the other reader and to the reference standard result. When the two readers gave discordant results, a third reader was consulted to resolve the discrepancy. The details of the tests, as well as their performance per origin of specimens in our evaluation, are presented elsewhere (14).

### Simulated algorithms.

Results of the RDTs performed at ITM were used to construct simulated algorithms using the WHO-recommended testing strategies for high (≥5%)-prevalence and low (<5%)-prevalence settings, as described for Fig. 1A and B. We could not perform simulations of the repetition of the tests for discordant RDT1^+^ RDT2^−^ results or retest 14 days later, as recommended by WHO. All simulations used RDT Determine as the first test. For RDT2 and RDT3, we selected all assays that met WHO recommendations, i.e., sensitivity of ≥99% and specificity of ≥99%, based on their individual performance estimates, compared to the reference algorithm, per origin of specimens (14). For sites where fewer than two tests met these criteria, we expanded the criteria to tests that had a specificity estimate of ≥98% or ≥96%. We also ensured that assays RDT2 and RDT3 had higher specificity than RDT1 in all the algorithms simulated here.

In addition, we simulated a testing strategy using an RDT as a screening test, followed by a simple confirmatory assay (Fig. 1C). For the screening test, we used all RDTs that met the WHO recommendations for the first assay, i.e., sensitivity of ≥99% and specificity of ≥98%.

### Statistical analysis.

STATA version 13.1 (StataCorp, College Station, Texas, USA) was used to carry out data analysis.

As for any performance evaluation, the results of the simulated algorithms were compared to those of the reference algorithm, considered the gold standard. We performed an inverse-probability weighted analysis to adjust for the initial sampling strategy, which underrepresented samples classified as negative by the onsite algorithm. For each participant, the weight was calculated as the inverse of the probability of inclusion in the study, i.e., as the total number of clients with similar onsite results during the study period divided by the number of included participants with similar results.

Since all tests included in this evaluation were antibody tests and were not expected to detect acute infections, we excluded samples classified as acute infections by the reference algorithm, i.e., positive with a fourth-generation EIA, negative or indeterminate with LIA, and positive with the antigen test (Fig. 1). We also excluded from all analyses samples with indeterminate results by the reference algorithm. Samples with an inconclusive result by a specific simulated algorithm were excluded from the estimates of sensitivity and specificity and predictive values of that specific algorithm, and their number and proportion are reported separately.

### Ethics.

The study was approved by the Médecins sans Frontières (MSF) Ethical Review Board and by ethics committees in the five countries where the samples were collected. All participants provided written informed consent.

## References

[1] World Health Organization. 2015 Consolidated guidelines on HIV testing services 2015. World Health Organization, Geneva, Switzerland.

[2] Shanks L, Klarkowski D, O'Brien DP 2013 False positive HIV diagnoses in resource limited settings: operational lessons learned for HIV programmes. PLoS One 8:e59906. doi:10.1371/journal.pone.0059906. 23527284 PMC3603939

[3] World Health Organization. 2004 Rapid HIV tests: guidelines for use in HIV testing and counselling services in resource-constrained settings. World Health Organization, Geneva, Switzerland http://apps.who.int/iris/handle/10665/42978.

[4] World Health Organization. 2012 Service delivery approaches to HIV testing and counselling (HTC): a strategic HTC programme framework. World Health Organization, Geneva, Switzerland http://www.who.int/hiv/pub/vct/htc_framework/en/.

[5] Kagulire SC, Opendi P, Stamper PD, Nakavuma JL, Mills LA, Makumbi F, Gray RH, Shott JP, Serwadda D, Reynolds SJ 2011 Field evaluation of five rapid diagnostic tests for screening of HIV-1 infections in rural Rakai, Uganda. Int J STD AIDS 22:308–309. doi:10.1258/ijsa.2009.009352. 21680664 PMC3726838

[6] Klarkowski D, Glass K, O'Brien D, Lokuge K, Piriou E, Shanks L 2013 Variation in specificity of HIV rapid diagnostic tests over place and time: an analysis of discordancy data using a Bayesian approach. PLoS One 8:e81656. doi:10.1371/journal.pone.0081656. 24282615 PMC3840056

[7] Gray RH, Makumbi F, Serwadda D, Lutalo T, Nalugoda F, Opendi P, Kigozi G, Reynolds SJ, Sewankambo NK, Wawer MJ 2007 Limitations of rapid HIV-1 tests during screening for trials in Uganda: diagnostic test accuracy study. BMJ 335:188. doi:10.1136/bmj.39210.582801.BE. 17545184 PMC1934458

[8] Lejon V, Ngoyi DM, Ilunga M, Beelaert G, Maes I, Büscher P, Fransen K 2010 Low specificities of HIV diagnostic tests caused by Trypanosoma brucei gambiense sleeping sickness. J Clin Microbiol 48:2836–2839. doi:10.1128/JCM.00456-10. 20573878 PMC2916589

[9] Singer DE, Kiwanuka N, Serwadda D, Nalugoda F, Hird L, Bulken-Hoover J, Kigozi G, Malia JA, Calero EK, Sateren W, Robb ML, Wabwire-Mangen F, Wawer M, Gray RH, Sewankambo N, Birx DL, Michael NL 2005 Use of stored serum from Uganda for development and evaluation of a human immunodeficiency virus type 1 testing algorithm involving multiple rapid immunoassays. J Clin Microbiol 43:5312–5315. doi:10.1128/JCM.43.10.5312-5315.2005. 16208006 PMC1248521

[10] Anzala O, Sanders EJ, Kamali A, Katende M, Mutua GN, Ruzagira E, Stevens G, Simek M, Price M 2008 Sensitivity and specificity of HIV rapid tests used for research and voluntary counselling and testing. East Afr Med J 85:500–504. 19537426 10.4314/eamj.v85i10.9666

[11] Shanks L, Siddiqui MR, Kliescikova J, Pearce N, Ariti C, Muluneh L, Pirou E, Ritmeijer K, Masiga J, Abebe A 2015 Evaluation of HIV testing algorithms in Ethiopia: the role of the tie-breaker algorithm and weakly reacting test lines in contributing to a high rate of false positive HIV diagnoses. BMC Infect Dis 15:39. doi:10.1186/s12879-015-0769-3. 25645240 PMC4331460

[12] Kroidl I, Clowes P, Mwalongo W, Maganga L, Maboko L, Kroidl AL, Geldmacher C, Machibya H, Hoelscher M, Saathoff E 2012 Low specificity of determine HIV1/2 RDT using whole blood in south west Tanzania. PLoS One 7:e39529. doi:10.1371/journal.pone.0039529. 22768086 PMC3387183

[13] Klarkowski D, O'Brien DP, Shanks L, Singh KP 2014 Causes of false-positive HIV rapid diagnostic test results. Expert Rev Anti Infect Ther 12:49–62. doi:10.1586/14787210.2014.866516. 24404993

[14] Kosack CS, Page A-L, Beelaert G, Benson T, Savane A, Ng'ang'a A, Andre B, Zahinda J-PB, Shanks L, Fransen K 2017 Towards more accurate HIV testing in sub-Saharan Africa: a multi-site evaluation of HIV RDTs and risk factors for false positives. J Int AIDS Soc 19:1–12. doi:10.7448/IAS.20.1.21345. PMC5467586 28364560

[15] UNAIDS. 2014 The Gap report. UNAIDS, Geneva, Switzerland http://www.unaids.org/en/resources/campaigns/2014/2014gapreport/gapreport.

[16] Kosack CS, Shanks L, Beelaert G, Benson T, Savane A, Ng'ang'a A, Andre B, Zahinda J-PBN, Fransen K, Page A-L 2017 HIV misdiagnosis in sub-Saharan Africa: performance of diagnostic algorithms at six testing sites. J Int AIDS Soc 20:1–18. doi:10.7448/IAS.20.1.21419. PMC5515032 28691437

[17] Baveewo S, Kamya MR, Mayanja-Kizza H, Fatch R, Bangsberg DR, Coates T, Hahn JA, Wanyenze RK 2012 Potential for false positive HIV test results with the serial rapid HIV testing algorithm. BMC Res Notes 5:154. doi:10.1186/1756-0500-5-154. 22429706 PMC3392728

[18] Crucitti T, Taylor D, Beelaert G, Fransen K, Van Damme L 2011 Performance of a rapid and simple HIV testing algorithm in a multicenter phase III microbicide clinical trial. Clin Vaccine Immunol 18:1480–1485. doi:10.1128/CVI.05069-11. 21752945 PMC3165239

[19] Galiwango RM, Musoke R, Lubyayi L, Ssekubugu R, Kalibbala S, Ssekweyama V, Mirembe V, Nakigozi G, Reynolds SJ, Serwadda D, Gray RH, Kigozi G 2013 Evaluation of current rapid HIV test algorithms in Rakai, Uganda. J Virol Methods 192:25–27. doi:10.1016/j.jviromet.2013.04.003. 23583487 PMC3749432

[20] Lyamuya EF, Aboud S, Urassa WK, Sufi J, Mbwana J, Ndugulile F, Massambu C 2009 Evaluation of simple rapid HIV assays and development of national rapid HIV test algorithms in Dar es Salaam, Tanzania. BMC Infect Dis 9:19. doi:10.1186/1471-2334-9-19. 19226452 PMC2650699

[21] Flynn D, Johnson C, Sands A, Wong V, Figueroa C, Baggaley R 2015 Uptake of WHO recommended HIV testing strategies: an analysis of national policies on HIV testing services. Poster World Health Organization, Geneva, Switzerland http://www.who.int/hiv/pub/posters/testing-strategies-uptake/en/.

[22] Klarkowski DB, Wazome JM, Lokuge KM, Shanks L, Mills CF, O'Brien DP 2009 The evaluation of a rapid in situ HIV confirmation test in a programme with a high failure rate of the WHO HIV two-test diagnostic algorithm. PLoS One 4:e4351. doi:10.1371/journal.pone.0004351. 19197370 PMC2633037

[23] Moon H-W, Huh HJ, Oh GY, Lee SG, Lee A, Yun Y-M, Hur M 2015 Evaluation of the Bio-Rad Geenius HIV 1/2 confirmation assay as an alternative to Western blot in the Korean population: a multi-center study. PLoS One 10:e0139169. doi:10.1371/journal.pone.0139169. 26422281 PMC4589337

[24] Mor O, Mileguir F, Michaeli M, Levy I, Mendelson E 2014 Evaluation of the Bio-Rad Geenius HIV 1/2 assay as an alternative to the INNO-LIA HIV 1/2 assay for confirmation of HIV infection. J Clin Microbiol 52:2677–2679. doi:10.1128/JCM.01184-14. 24789189 PMC4097735

[25] Montesinos I, Eykmans J, Delforge M-L 2014 Evaluation of the Bio-Rad Geenius HIV-1/2 test as a confirmatory assay. J Clin Virol 60:399–401. doi:10.1016/j.jcv.2014.04.025. 24932737

[26] Malloch L, Kadivar K, Putz J, Levett PN, Tang J, Hatchette TF, Kadkhoda K, Ng D, Ho J, Kim J 2013 Comparative evaluation of the Bio-Rad Geenius HIV-1/2 confirmatory assay and the Bio-Rad Multispot HIV-1/2 rapid test as an alternative differentiation assay for CLSI M53 algorithm-I. J Clin Virol 58(Suppl 1):e85–e91. doi:10.1016/j.jcv.2013.08.008. 24342484

[27] Hawthorne Hallen A, Samuelson A, Nordin M, Albert J, Bogdanovic G 2014 Evaluation of bio-rad Geenius HIV-1 and -2 assay as a confirmatory assay for detection of HIV-1 and -2 antibodies. Clin Vaccine Immunol 21:1192–1194. doi:10.1128/CVI.00153-14. 24943380 PMC4135904

[28] Abbate I, Pergola C, Pisciotta M, Sciamanna R, Sias C, Orchi N, Libertone R, Ippolito G, Capobianchi MR 2014 Evaluation in a clinical setting of the performances of a new rapid confirmatory assay for HIV1/2 serodiagnosis. J Clin Virol 61:166–169. doi:10.1016/j.jcv.2014.06.015. 25037532

[29] Herssens N, Beelaert G, Fransen K 2014 Discriminatory capacity between HIV-1 and HIV-2 of the new rapid confirmation assay Geenius. J Virol Methods 208:11–15. doi:10.1016/j.jviromet.2014.07.025. 25075934

[30] Plate DK, Rapid HIV Test Evaluation Working Group. 2007 Evaluation and implementation of rapid HIV tests: the experience in 11 African countries. AIDS Res Hum Retroviruses 23:1491–1498. doi:10.1089/aid.2007.0020. 18160006

